# Association of *BMPR1A* polymorphism, but not *BMP4*, with kidney size in full-term newborns

**DOI:** 10.1007/s00467-012-2277-7

**Published:** 2012-08-11

**Authors:** Mariusz Kaczmarczyk, Iwona Goracy, Beata Loniewska, Anna Kuprjanowicz, Agnieszka Binczak-Kuleta, Jeremy S. Clark, Andrzej Ciechanowicz

**Affiliations:** 1 Department of Clinical and Molecular Biochemistry, Pomeranian Medical University, ul. Powstancow Wlkp. 72, 70-111 Szczecin, Poland; 2 Department of Neonatal Diseases, Pomeranian Medical University, ul. Powstancow Wlkp. 72, 70-111 Szczecin, Poland; 3 Department of Radiology, Pomeranian Medical University, ul. Powstancow Wlkp. 72, 70-111 Szczecin, Poland

**Keywords:** Bone morphogenetic proteins, Gene polymorphism, Nephron number, Newborns, Branching morphogenesis

## Abstract

**Background:**

A correlation between renal mass and nephron number in newborns allows the use of total kidney volume at birth as a surrogate for congenital nephron number. As the bone morphogenetic protein type 4 (BMP4), and its receptor type 1A (BMPR1A, ALK3), play an important role in renal development, we hypothesized that common, functional polymorphisms in their genes might be responsible for variation in kidney size among healthy individuals.

**Methods:**

We recruited 179 healthy full-term newborns born to healthy women. Kidney volume was measured sonographically. Total kidney volume (TKV) was calculated as the sum of left and right kidneys, and normalized for body surface area (TKV/BSA). Genomic DNA was extracted from umbilical cord blood leukocytes, and c.455T > C (rs17563) *BMP4* and c.67 + 5659A > T (rs7922846) *BMPR1A* genotypes were identified by PCR-RFLP.

**Results:**

TKV/BSA in newborns carrying at least one A *BMPR1A* allele (AA + AT) was significantly reduced by approximately 13 % as compared with TT homozygous newborns (106.7 ± 21.5 ml/m^2^ vs. 122.7 ± 43.8 ml/m^2^, *p* < 0.02). No significant differences in TKV/BSA were found among newborns with different *BMP4* genotypes.

**Conclusions:**

Results suggest that rs7922846 *BMPR1A* polymorphism may account for subtle variation in kidney size at birth, reflecting congenital nephron endowment.

## Introduction

Suboptimal nephron number may be associated with increased risk for essential hypertension and susceptibility to renal injury [1–4], but the factors that set nephron number during kidney development remain largely undefined [5]. Nonetheless, genetic studies in humans and mice have provided valuable insights into the possible genetic contribution and molecular mechanisms leading to normal nephron endowment and renal hypoplasia [2]. In humans, final nephron endowment for life is determined during late gestation and displays wide individual variation (210,332 to 2,702,079 nephrons per kidney) [6–8]. El Kares et al. hypothesized that congenital nephron number is a multifactorial trait controlled by the interaction of environmental factors and genetic variants that influence the extent of branching nephrogenesis during fetal life [9]. In addition, confirmation of a strong correlation between renal mass and nephron number in newborns allows the use of the total kidney volume at birth (measured by ultrasonography) as a surrogate for congenital nephron number [10].

Bone morphogenetic proteins (BMPs) belong to the Transforming Growth Factor-β (TGF-β) family, with more than 30 members that bind to two types of serine-threonine kinase receptors, i.e., type 1 (BMPR1) and type 2 (BMPR2) receptors [11]. Although BMPs were first identified as factors that induce the formation of bone and cartilage when implanted at ectopic sites in rats [11], they are now known to be implicated in a variety of developmental processes [12]. The spatial and temporal expression of Bone Morphogenetic Protein type 2 or type 4 (BMP2 or BMP4, respectively) and their shared receptor, BMP receptor type 1A (BMPR1A)—also known as Activin-Like Kinase 3 (ALK3) [11]—suggests a functional role for BMP signaling during the formation of intermediate mesoderm-derived organs, including the mammalian kidney [13]. In 2008, Weber et al. identified *BMP4* mutations causing renal hypodysplasia, characterized by a reduction in nephron number and a small overall kidney size [14]. Recently, Di Giovanni et al. reported that *Alk3*-deficient mice exhibit simple renal hypoplasia characterized by decreases in both kidney size and nephron number, but with normal tissue architecture [13]. In 2009, Capasso et al. [15] found that cutaneous melanoma-associated c.455T > C (rs17563) *BMP4* polymorphism, resulting in an amino acid change from valine to alanine at residue 152 (p.Val152Ala), affects *BMP4* gene expression, and Boettcher et al. suggested that the obesity-associated c.67 + 5659A > T (rs7922846) *BMPR1A* polymorphism modulates *BMPR1A* mRNA levels [16].

Thus far, several common polymorphisms in developmental genes including *PAX2* [5], *RET* [10], *ALDH1A2* [9], *OSR1* [17], and *ACE* [18] have been associated with subtle variation in kidney size at birth. These findings lend support to the hypothesis that common, functional polymorphisms in *BMP4* and/or *BMPR1A* may be responsible for the variation in nephron number that is seen amongst healthy individuals. To verify this hypothesis, we examined the association of nonsynonymous rs17563 *BMP4* and intronic rs7922846 *BMPR1A* polymorphisms with congenital kidney volume, a surrogate measure of the number of nephrons, in a cohort of healthy, full-term newborns in Poland.

## Methods

### Study subjects

At the Department of Neonatal Diseases at the Pomeranian Medical University in Szczecin, we prospectively recruited 179 consecutive healthy Polish newborns (79 females and 100 males), born after the end of the 37th week of gestation to healthy women with uncomplicated pregnancies. All children were breast-fed and free of medication. Twins, infants of mothers with preeclampsia, hypertension of any cause, diabetes, history of illicit substance use, or antenatal steroid therapy were excluded. Other exclusion criteria were congenital infection, intra-uterine growth restriction (i.e., below the 10th percentile birth mass, length, or head circumference), chromosomal aberrations or congenital malformations. At birth, cord blood of newborns was obtained for isolation of genomic DNA. The gender of the newborn, birth mass (BM), and birth length (BL) were taken from standard hospital records. Body surface area (BSA) was calculated as the square root of [BL (cm) × BM (kg)/3600] according to Mosteller [19]. The study was approved by the local ethics committee and parents gave informed consent.

### Kidney volume measurement

Sonographic examination was performed in newborns on the third day after delivery with an EnVisor C machine (Philips Canada, Markham, Ontario, Canada) using a 5-MHz sector probe (Philips Canada) and 10-MHz linear probe (Philips Canada) as described previously [20, 21]. Left and right kidney volumes (LKV and RKV, respectively) were calculated using the following formula for volume of an ellipsoid: $\left[ {{\text{kidney}}\,{\text{volume}} = \left( {{{4} \left/ {3} \right.}} \right) \times {\text{Pi}} \times \left( {{{\text{length}} \left/ {{2}} \right.}} \right) \times \left( {{{\text{height}} \left/ {{2}} \right.}} \right) \times \left( {{{\text{width}} \left/ {2} \right.}} \right)} \right]$ [5]. Total kidney volume (TKV) was calculated as the sum of LKV and RKV. Subsequently, TKV was normalized for body surface area (TKV/BSA) [5, 20].

### Determination of *BMP4* and *BMPR1A* genotypes

Genomic DNA from cord blood was isolated with the QIAamp Blood DNA Mini Kit (QIAGEN, Germany). For the analysis of the rs17563 *BMP4* gene polymorphism, a polymerase chain reaction/restriction fragment length polymorphism (PCR/RFLP) method was designed with the following primer pair: forward: 5′-ACTCTgCTTTTCgTTTCCTCTTTA-3′ and reverse 5′-ggCCCAATTCCCACTCC-3′ primers (TIB MOL BIOL, Poznań, Poland). The *BMP4* amplicons were subsequently digested with *Hph*I enzyme (MBI Fermentas, Vilnius, Lithuania). The PCR *BMP4* product of 395 base pairs (bp) was cut into fragments of 244, 75, 62, and 14 bp from the T allele and 306, 75, and 14 bp from the C allele.

DNA fragments that contained the rs7922846 *BMPR1A* polymorphism were amplified by PCR with forward 5′-TTTCAgCGCTCAATAgACAC-3′ and reverse 5′-TCCCTCCCCCTTTCATA-3′ primers. PCR-RFLP with the *Acc*I restriction enzyme was performed. In the case of the T allele, the final product of 541 bp remained undigested, while the A variant gave restriction fragments of 352 bp and 189 bp. The restriction fragments in each case were electrophoretically separated and visualized in ethidium bromide-stained 3 % agarose gels.

### Statistical analysis

Possible divergence of *BMP4* or *BMPR1A* genotype frequencies from Hardy-Weinberg equilibrium was assessed using a χ^2^ test. The distribution of each quantitative variable was tested for skewedness. Quantitative data were presented as means ± SD. Association of either gender or genotype (with respect to dominant and recessive modes of inheritance of the minor alleles) with each outcome variable was assessed by Student’s *t* test using STATISTICA (StatSoft, Inc. (2011), version 10). Statistical significance was defined as *p* < 0.05.

## Results

All PCR samples were genotyped twice and a concordance rate of 100 % was attained. There were 57 CC *BMP4* homozygotes (31.8 %), 93 CT heterozygotes (52.0 %), and 29 TT homozygotes (16.2 %). There were 82 AA *BMPR1A* homozygotes (45.8 %), 81 AT heterozygotes (45.3 %), and 16 TT homozygotes (8.9 %). The frequencies of the minor alleles were: 42.2 % (39.5 % in newborn males and 45.6 % in newborn females) and 31.6 % (33.0 % in newborn males and 29.7 % in newborn females) for T *BMP4* and T *BMPR1A*, respectively.

Both *BMP4* and *BMPR1A* genotype distributions conformed to the expected Hardy-Weinberg equilibrium (*p* = 0.545 and *p* = 0.607, respectively). Characteristics of the newborn cohort (*n* = 179) with respect to gender are shown in Table 1.

**Table 1:** Characteristics of newborn cohort with respect to gender

Variable	Total (*n* = 179)	Males (*n* = 100)	Females (*n* = 79)	*p* ^a^
Gestational age (weeks)	39.5 ± 1.3	39.5 ± 1.2	39.4 ± 1.4	0.711
BM (kg)	3.47 ± 0.45	3.55 ± 0.48	3.37 ± 0.37	0.007
BL (m)	0.56 ± 0.03	0.56 ± 0.03	0.56 ± 0.03	0.334
BSA (m^2^)	0.232 ± 0.019	0.235 ± 0.021	0.228 ± 0.016	0.018
LKV (ml)	12.9 ± 3.4	13.3 ± 3.5	12.5 ± 3.1	0.121
RKV (ml)	12.1 ± 3.3	12.5 ± 3.5	11.7 ± 3.0	0.102
LKV/RKV	1.09 ± 0.24	1.09 ± 0.28	1.09 ± 0.20	0.853
TKV (ml)	25.0 ± 6.1	25.7 ± 6.3	24.1 ± 5.6	0.079
TKV/BSA (ml/m^2^)	108.2 ± 24.6	109.7 ± 25.1	106.2 ± 24.0	0.338

*BM* body mass, *BL* birth length, *BSA* body surface area, *LKV* left kidney volume, *RKV* right kidney volume, *TKV* total kidney volume

^a^Males vs. females

The distribution of these characteristics in our cohort, including kidney size, approached normality (skewedness < 2 for all variables). The mean values of birth mass and BSA in male newborns were significantly higher as compared with female newborns. No significant differences in gestational age, birth length, LKV, RKV, LKV/RKV, TKV, and TKV/BSA were found between male and female newborns.

Characteristics of kidney volumes with respect to rs17563 *BMP4* and rs7922846 *BMPR1A* polymorphisms are shown in Table 2. Mean values of TKV and TKV/BSA in newborns with at least one A allele (AA + AT) were significantly lower as compared with TT *BMPR1A* homozygotes (24.7 ± 5.5 ml versus 28.0 ± 10.0 ml and 106.7 ± 21.5 ml/m^2^ versus 122.7 ± 43.8 ml/m^2^, respectively). No significant differences in LKV, RKV, LKV/RKV ratio were found among newborns with different *BMPR1A* genotypes. No significant differences in LKV, RKV, LKV/RKV ratio, TKV, and TKV/BSA were found among newborns with different *BMP4* genotypes.

**Table 2:** Characteristics of kidney volumes in newborns with respect to *BMP4* and *BMPR1A* polymorphisms

Variable	*BMPR1A* (A5659T, rs7922846)			p_D_^a^	p_R_^a^	*BMP4* (T455C, rs17563)			p_D_^a^	p_R_^a^
	AA (*n* = 82)	AT (*n* = 81)	TT (*n* = 16)			CC (*n* = 57)	CT (*n* = 93)	TT (*n* = 29)		
LKV (ml)	12.8 ± 3.4	12.8 ± 2.9	14.4 ± 4.8	0.951	0.062	12.7 ± 3.0	13.0 ± 3.6	12.9 ± 3.4	0.560	0.951
RKV (ml)	11.7 ± 3.0	12.3 ± 3.0	13.6 ± 5.4	0.247	0.070	11.8 ± 2.7	12.1 ± 3.4	12.8 ± 4.0	0.429	0.247
LKV/RKV	1.1 ± 0.3	1.1 ± 0.2	1.1 ± 0.2	0.484	0.947	1.1 ± 0.2	1.1 ± 0.3	1.1 ± 0.3	0.858	0.484
TKV (ml)	24.4 ± 5.8	25.1 ± 5.1	28.0 ± 10.0	0.505	0.043	24.5 ± 5.3	25.1 ± 6.4	25.7 ± 6.6	0.449	0.505
TKV/BSA (ml/m^2^)	105.7 ± 23.3	107.8 ± 19.8	122.7 ± 43.8	0.327	0.013	106.1 ± 20.9	108.1 ± 25.0	112.3 ± 30.1	0.457	0.327

*LKV* left kidney volume, *RKV* right kidney volume, *TKV* total kidney volume, *BSA* body surface area

^a^p_D_ and p_R_ are test probabilities with dominant (TT + AT versus AA [rs7922846], TT + CT versus CC [rs17563]) and recessive (TT versus AT + AA [rs7922846], TT versus CT + CC [rs17563]) modes of inheritance of the minor alleles, respectively

## Discussion

The present study demonstrates for the first time an association of rs7922846 (c.67 + 5659A > T) *BMPR1A* polymorphism, but not rs17563 (c.455 T > C) *BMP4*, with kidney size in Polish full-term newborns. Total kidney volume, standardized for body surface area (TKV/BSA), in newborns carrying at least one A *BMPR1A* allele (AA + AT) was reduced by approximately 13 % as compared with TT homozygous newborns (a recessive genetic model for the T allele). The number of nephrons formed during fetal organogenesis seems to be an important determinant of human health, and reduced nephron endowment may result in pathological states ranging from early renal failure to adult-onset hypertension, depending on the severity of nephron deficiency [1–4].

We found no relationship between rs7922846 and kidney volume under the assumption of dominant genetic model for the T allele (TT + AT versus AA). Therefore, our results support a dominance of the A *BMPR1A* allele, which is evidenced in the similarity of kidney volumes, TKV/BSA in particular, in carriers of at least one A allele. Recently, Böttcher et al. [16] reported a significant association between rs7922846 variant and mRNA expression of *BMPR1A* only in the dominant model for the T allele (TT + AT versus AA). The recessive model, however, was not calculated due to the small sample size of the groups of subjects homozygous for the T allele [16]. Further research may be needed to determine which genetic model best matches the actual underlying mode of inheritance of the A allele, associated with reduced total kidney volume.

Although the TKV/BSA was significantly associated with *BMPR1A* variant, the effect of this polymorphism was less evident when individual kidneys were evaluated. We previously showed that the common angiotensin converting enzyme (ACE) insertion/deletion (I/D) polymorphism was not only associated with the TKV/BSA but also with individual kidney volumes [18]. While the ACE DD homozygotes accounted for around 25 % of all genotypes in the aforementioned study [18], the frequency of *BMPR1A* TT homozygotes in the present study was 8.9 %. Therefore, the low statistical power due to insufficient sample size may have led to borderline significance for individual, left and right kidneys.

The intermediate mesoderm, differentiating into the metanephric blastema (and then metanephric mesenchyme) and ureteric bud [22], gives rise to the permanent kidney, but extrinsic signals altering gene expression are required to commit the intermediate mesoderm to a renal lineage [13]. James and Schultheiss showed that BMP-ALK signaling modulates gene expression patterns in the intermediate mesoderm in vitro and in vivo [23]. BMPR1A (ALK3) is one of the three type 1 BMP (bone morphogenetic protein) receptors that are essential for BMP signaling [24]. Böttcher et al. [16] showed that non-diabetic homozygous carriers of the obesity risk alleles for rs7922846 (c.67 + 5659A) and three other *BMPR1A* variants (rs7095025, rs11202222, and rs10788528) had higher visceral and subcutaneous adipose tissue mRNA expression of *BMPR1A*, yet only rs7922846 was significantly associated with increased visceral mRNA expression [16]. Moreover, this polymorphism is in strong linkage disequilibrium with another nonsynonymous rs11528010 *BMPR1A* polymorphism causing the amino acid substitution of proline by threonine at residue 2 (p.Pro2Thr) located in the potential BMPR1A signal peptide chain that could affect posttranslational transport of BMPR1A [16].

In our study, carriers of the A allele of *BMPR1A* polymorphism, associated with increased ALK3 mRNA expression, had significantly smaller kidney size as compared with TT homozygous newborns. Animal studies show that both decreased and increased expression of active ALK3 receptor may ultimately result in deficient UB branching [25, 26]. Although both ALK3 loss-of-function and gain-of-function mutation mouse models exhibit similar outcomes, the mechanisms underlying these outcomes are distinct [26]. While ALK3 deficiency disrupts UB patterning—an increased number of first and second UB branches followed by a decrease in the number of subsequent branches formed—resulting in an overall reduction in UB number, the constitutive overexpression of ALK3 inhibits branching morphogenesis [26]. As even minor defects of UB branching can result in a marked decrease in nephron number [27], we thus hypothesize that the reduced kidney size seen in healthy newborns carrying at least one A *BMPR1A* allele might be due to subtle decreases in the efficiency of UB branching, possibly mediated by increased expression of *BMPR1A* mRNA [16]. However, whether expression of *BMPR1A* gene mRNA level correlates with protein abundance remains to be determined.

Bone morphogenetic protein 4 (BMP4) has been implicated in several aspects of embryonic development, especially in those organs in which epithelial–mesenchymal interactions are essential for development, such as the kidney [28, 29]. Additionally, it is a known regulator of UB branching; firstly, by inhibiting ectopic budding from the nephric (Wolffian) duct and, secondly, by promoting elongation of the branching ureter [30]. Previously, BMP signaling has been shown to regulate nephron number in mice, as deletion of the Cv2 molecule, which normally has pro-BMP function, resulted in reduced nephron endowment [31]. Although three missense mutations in *BMP4* have been recently detected in children with renal hypodysplasia (RHD), characterized by reduced kidney size and/or maldevelopment of the renal tissue [14], we failed to detect an association between rs17563 *BMP4* polymorphism and newborn kidney size. There was, however, a trend towards lower individual and total kidney volumes in carriers of the CC genotype. Recently, Cappaso et al. [15] reported significantly lower *BMP4* mRNA levels in CC homozygotes as compared to carriers of the T allele. Thus, the study cohort might be too small to definitively exclude effects of this BMP4 variant. However, if the lack of association is a true negative result, this finding seems to be supported by Cain and Bertram [29], who showed that despite reduced endogenous *BMP4* mRNA levels, most *BMP4* heterozygous mouse embryos were still able to facilitate normal ureteric branching morphogenesis during development. Although many mutations within *BMP4* leading to various phenotypes are known [32], to our knowledge, rs17563 is the only identified polymorphism in the coding region [33]. As the rs17563 *BMP4* polymorphism was previously found to be significantly associated with *BMP4* mRNA levels and BMP4 plasma levels [15], we also tried to account for possible ligand-dependent (BMP4) up- or down-regulation of BMPR1A. To do this, we analyzed TKV values for compound genotypes composed of *BMP4* and *BMPR1A*, but no associations were found (data not shown).

In order to minimize the effects of potential confounding variables that could create a spurious association, the study was conducted in a carefully selected group of newborns all of whom met all criteria for inclusion. The sonographic examinations of kidney size were performed according to protocols in the literature that give reproducible results, and kidney volumes were similar to those of a large cohort of Danish newborns who were studied within the first 5 days of life [20]. The *BMPR1A* genotype distribution in our group was in accordance with Hardy-Weinberg equilibrium. TKV/BSA in infants with at least one A *BMPR1A* allele ranged from 54.5 to 173.2 ml/m^2^ versus 69.2 to 219.3 ml/m^2^ in TT homozygotes, and the left/right kidney volume ratio did not differ among newborns with the various *BMPR1A* genotypes. Therefore, it is wrong to assume that the significant association is due to a few newborns with very small kidneys or with unilateral renal hypoplasia.

The potential weakness of the present study stems from the fact that our sample was not systematically tested for genetic heterogeneity. Population stratification is a concern in both case-control and cross-sectional studies [34]. Moreover, Berger et al. showed that population substructures can be detected even in an apparently homogenous population [35]. The confounded association resulting from stratification or admixture within a population can be reduced by matching by geographical region and/or by markers of ethnic origin [36]. The population now inhabiting the region of West Pomerania resulted from extensive mixing of Polish people from all regions of Poland after the Second World War and therefore can provide a representative sample of a Caucasian-European population [37]. In addition, the rs7922846 allele frequencies were similar to those reported in adult control subjects in a Leipzig cohort, a self-contained Sorbian population in Germany [16], and Utah residents with ancestry from Northern and Western Europe from the HapMap project [38]. Although, we believe, it is very unlikely that our cohort of newborn infants is a biased sample due to population stratification, other studies in populations of the highest possible level of homogeneity may be needed.

In summary, our data suggest that common genetic variation in *BMPR1A* may play a role in early human nephrogenesis, through putative increase in *BMPR1A* mRNA expression and thereby, presumably, defective ureteric bud branching leading to subtle reduction in nephron number and subsequently to slightly smaller kidney size in healthy newborns. Furthermore, rs7922846 *BMPR1A* polymorphism may, in concert with other common polymorphisms in developmental genes including *PAX2* [5], *RET* [10], *ALDH1A2* [9], *OSR1* [17] or *ACE* [18], partially account for subtle variation in kidney size at birth reflecting congenital nephron endowment. Thus, newborns possessing an unfavorable polygenic profile associated with reduced nephron number may benefit from heightened surveillance for essential hypertension in adulthood.
